# A New Omni-Directional EMAT for Ultrasonic Lamb Wave Tomography Imaging of Metallic Plate Defects

**DOI:** 10.3390/s140203458

**Published:** 2014-02-20

**Authors:** Songling Huang, Zheng Wei, Wei Zhao, Shen Wang

**Affiliations:** State Key Lab of Power Systems, Department of Electrical Engineering, Tsinghua University, Beijing 100084, China; E-Mails: weizhengbj@163.com (Z.W.); zhaowei@mail.tsinghua.edu.cn (W.Z.); wangshen@tsinghua.org.cn (S.W.)

**Keywords:** omni-directional EMAT, contra-flexure coil, ultrasonic Lamb wave, tomography imaging, metallic plate defects, projection data extracting

## Abstract

This paper proposes a new omni-directional electromagnetic acoustic transducer (EMAT) for the ultrasonic Lamb wave (ULW) tomography imaging (TI) of defects in metallic plates. The proposed EMAT is composed of a permanent magnet and a coil with a contra-flexure structure. This new EMAT coil structure is used for omni-directional ULW transmission and reception and ULW TI for the first time. The theoretical background and the working principles of this EMAT are presented and analyzed. The experimental results of its use on a 3 mm thick aluminum plate indicate that the EMAT with a contra-flexure coil (CFC) can transmit and receive a pure single A0 mode ULW with a high signal-to-noise ratio (SNR). Thus, the extraction of the projection data used for ULW TI may be performed accurately. The circumferential consistency of the projection data is only slightly influenced by the distortion of the eddy current field that is induced by the new CFC with an irregular shape. When the new EMAT array is used for ULW TI using the cross-hole method and SIRT arithmetic, a desirable imaging quality can be achieved, and the estimated size of an artificial corrosion defect agreed well with its actual value. The relation between the reconstruction resolution and the number of the new EMATs used is analyzed. More TI experiments are carried out when the aluminum plate defect is in two different locations relative to the EMAT array, for the further investigation of the performances of the new EMATs.

## 1. Introduction

Metallic plates are widely used in the aerospace and automobile industries as well as in pressure vessels. During production and utilization, these metallic plates will inevitably acquire a number of defects that can cause serious, adverse influences on the safe service of the facilities and equipment made from them. Therefore, these metallic plates must be timely and efficiently inspected. Relative to the traditional inspection methods of point-by-point scanning, ultrasonic Lamb wave (ULW) inspection technology allows for a line with long distance to be scanned after a single excitation. Because the ULW inspection technology also has the advantages of being fast and efficient during an entire volumetric inspection [1–4], it is suitable for the detection and diagnosis of defects in metallic plates. Recently, studies on ULW inspection have not only focused on discovering these defects, but they have also attempted to accurately determine the size and shape of the defects. The technology of ULW tomography imaging (TI), which is derived from medical X-ray computed tomography, is one of the most effective ways of collecting detailed information about the defects in metallic plates [5]. It utilizes an array of transducers to transmit and receive a group of ULW rays from all directions. In this way, the inspected area of the plates can be scanned from different angles to generate the projection data. Next, the image of the inspected area can be reconstructed using these projection data, and the sizes and shapes of the defects in the inspected area can be accurately determined [6].

ULW TI produces quantitative maps of defects in metallic plates with different kinds of projection data, reconstruction algorithms and transducer array geometries. The time of flight (TOF), which is the time the ULW pulse takes to propagate from the transmitter to the receiver, the amplitude attenuation and the shift in the centroid frequency of the ULW are the most commonly used projection data for ULW TI [7–9]. In early studies, there were generally two groups of reconstruction algorithms for ULW TI. One group is the series expansion methods that use algebraic reconstruction techniques (ARTs) [10] and simultaneous iterative reconstruction techniques (SIRTs) [11]. The other group is the Fourier-based methods like filtered back projection (FBP) [7,8,12] and interpolated filtered back projection (IFBP) [13]. The geometries of the transducer arrays include the circular array configuration with parallel or fan beam projection [14], the linear array configuration such as cross-hole [10] and double cross-hole [15] and so on. In recent studies, a subset of the reconstruction algorithms of ULW TI, namely reconstruction algorithm for probabilistic inspection of damage (RAPID), has received great attention [16–19]. It generates maps of defects in terms of a presence probability using only a handful of transducers, and uses the feature of signal difference coefficient (SDC) as the projection data. Wang *et al.* [20–23] introduced virtual sensing paths and digital damage fingerprints into the RAPID-based ULW TI to improve the reconstruction performances. However, due to the sparse transducer arrays, the maps generated by RAPID-based TI may not offer accurate defect sizing or shape information, and will often produce false-positive artifacts [15]. Besides, some other recent studies have resulted in many different reconstruction techniques in an attempt to improve the resolution of ULW TI and reduce computational requirements or the number of the transducers required [24–28].

In practical applications, when metallic plates with large surface are scanned by transducer array using the ULW TI technology, TI are implemented by the transducer array at a regular interval. To scan and image the large surface plates fast and expediently, in every time of TI, the transducers are required to transmit and receive the ULW from all directions to collect the projection data from different angles. Thus, research on omni-directional ULW transducers for TI has become a highly studied area in this research field. Most of the traditional omni-directional ULW transducers for TI, such as piezoelectric transducers [19,29], can only be used to implement contacting inspection and are unable to complete the rapid scanning of large surfaces metallic plates [30]. In the literature, there are two main groups of non-contacting, omni-directional ULW transducers used for TI: micromachined silicon air-coupled capacitance transducers (MSACTs) and electromagnetic acoustic transducers (EMATs). Wright *et al.* [7] used MSACTs that consisted of contoured conducting backplates, silicon wafers and metalized Mylar films to image a non-circular defect and a group of multiple defects in a 0.69 mm thick aluminum plate using the ULW TI method. In their study, they used the attenuation and the shift in the centroid frequency of the ULW signals as the projection data. However, MSACTs place significant requirements on their manufacture and the environment, and the ultrasonic wave generated in the air by the MSACT will has a significant loss of energy when it propagates through the air-solid interface and the air gap between the MSACT and the inspected plate. Therefore, applications of MSACTs are limited [31]. Meanwhile, an EMAT directly generates the ultrasonic wave in the metallic plate based on the Lorentz force or the magnetostrictive effect such that the above energy loss does not exist. Moreover, the EMAT has a simple structure and a low cost. Hutchins *et al.* [8] used a pulsed laser source and an EMAT detector to image an 8 mm diameter through-hole defect in a 0.68 mm thick aluminum plate using the ULW TI method. In their study, the TOF, the attenuation and the shift in the centroid frequency of the ULW were used as the projection data. However, the ULW is characterized by multiple modes and a frequency dispersion, and the tone-burst pulse signal used to excite the EMAT contains numerous harmonic components. Thus, there exist numerous different ULW modes in the inspection result that cause the waveform detected by the EMAT to contain a number of interference signals. These interference signals severely disrupt the identification of the ULW mode of interest, influence the accuracy of the projection data extraction and reduce the quality of the ULW TI results.

A method to solve the above problems involves considering only certain frequency components of the ULW using a time-frequency analysis [15,32,33]. In 2007, Ho *et al.* [14] used an EMAT that consisted of a permanent magnet and a close-winding spiral coil (CWSC) to transmit and receive the ULW and a wavelet transform (WT) to identify and quantify the ULW signals for different modes. Next, the experimental ULW TI results for 0.69, 1.25 and 0.79 mm thick plates indicated that the accuracy of the projection data extraction and the imaging quality could be improved by using this method. However, during the process of scanning and imaging metallic plates with large surface, every ULW TI requires hundreds of projections, and every projection waveform needs to be processed using a WT to extract the projection data. This will requires a huge number of calculations and a large amount of work. The techniques of three-dimensional (3D) elastodynamic finite integration simulations are recently used to identify the multiple modes of ULW [34–36]. However they are still not practical to generate a full catalogue of possible defects in this way to collect the detail information about the real defects, because the computational requirements of the 3D behavior are large even for the defects with relatively simple geometries.

To overcome the above drawbacks, an omni-directional EMAT with a new structure is proposed in this paper. It has the advantages of the traditional omni-directional EMAT, and can control the mode of the generated ULW to make the inspected waveform easy to understand. The contra-flexure coil (CFC) is used as the transmitting coil and receiving coil for the newly proposed EMAT (hereafter referred to as the new EMAT). This new EMAT coil structure is used for omni-directional ULW transmitting and receiving and ULW TI for the first time. The theoretical background and the working principles of the new EMAT are presented and analyzed. Using the results of experiments on a 3 mm thick aluminum plate, the ability to transmit and receive the pure single A0 mode ULW of the new EMAT is verified. The accuracy of the projection data extraction, the circumferential consistency of the projection data as well as the feasibility and performances of being used for ULW TI of the new EMAT are further investigated experimentally.

## 2. Structural Design of the New Omni-Directional EMAT for ULW TI

### 2.1. Theoretical Background

**Figure 1.**
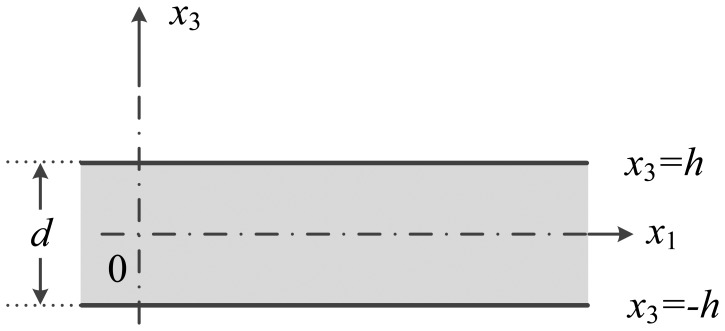
The sectional model of the free aluminum plate in space.

First of all, before the design of the new EMAT, the propagation characteristics of the ULW in the aluminum plates and the ideal operation point of the new EMAT should be analyzed to ensure that this new omni-directional EMAT has an acceptable performance for ULW TI. As TI only requires information about the ULWs that propagate from the transmitter to the receiver, the propagation distance is short. Thus, to simplify the model, the ULW attenuation is ignored during propagating in this paper. The sectional model of the free aluminum plate in the space is shown as Figure 1. The plate thickness *d* equals 2*h*. The line where *x*_3_ = ±*h* is the section line of the free surface of the free plate.

When the ultrasonic wave generated in the plate propagates to the top or bottom free surfaces, a complicated transforming of wave types occurs, and superposition and mutual interference occur among the different types of ultrasonic waves. Eventually, a stabilized ULW is formed, which propagates along the *x*_1_ direction. The ULW wave equations in the free aluminum plates are:

(1) $$\frac{\partial^{2}\phi }{\partial x_{1}^{2}}+\frac{\partial^{2}\phi }{\partial x_{3}^{2}}=\frac{1}{c_{L}^{2}}\frac{\partial^{2}\phi }{\partial t^{2}}$$

(2) $$\frac{\partial^{2}\varphi }{\partial x_{1}^{2}}+\frac{\partial^{2}\varphi }{\partial x_{3}^{2}}=\frac{1}{c_{T}^{2}}\frac{\partial^{2}\varphi }{\partial t^{2}}$$

where *c*_L_ and *c*_T_ are the velocity of the longitudinal wave and the transverse wave in the free aluminum plate. By solving Equations (1) and (2), the expressions of the displacements along the *x*_1_ and *x*_3_ direction in the aluminum plate induced by the ULW are determined as [37]:

(3) $$\{\begin{array}{l} u_{2}(x_{1},x_{3},t)=[ikA_{2}\cos(px_{3})+qB_{1}\cos(qx_{3})+ikA_{1}\sin(px_{3})-qB_{2}\sin(qx_{3})]\times \exp[i(kx_{1}-\omega t)]\\ u_{3}(x_{1},x_{3},t)=[-pA_{2}\sin(px_{3})-ikB_{1}\sin(qx_{3})+pA_{1}\cos(px_{3})-ikB_{2}\cos(qx_{3})]\times \exp[i(kx_{1}-\omega t)] \end{array}$$

where *A*_1_, *A*_2_, *B*_1_ and *B*_2_ are constants and *p*^2^ = *ω*^2^/*c*_L_^2^ − *k*^2^ and *q*^2^ = *ω*^2^/*c*_T_^2^ − *k*^2^. *k* is the ULW wave number and *ω* is the ULW angular frequency, which equals 2π*f*. According to the solving process of Equations (1) and (2), Equation (3) can be decomposed into two groups of solutions for the different modes:

(4) $$\{\begin{array}{l} u_{1}(x_{1},x_{3},t)=[ikA_{2}\cos(px_{3})+qB_{1}\cos(qx_{3})]\times \exp[i(kx_{1}-\omega t)]\\ u_{3}(x_{1},x_{3},t)=[-pA_{2}\sin(px_{3})+ikB_{1}\sin(qx_{3})]\times \exp[i(kx_{1}-\omega t)] \end{array}$$

(5) $$\{\begin{array}{l} u_{1}(x_{1},x_{3},t)=[ikA_{1}\sin(px_{3})-qB_{2}\sin(qx_{3})]\times \exp[i(kx_{1}-\omega t)]\\ u_{3}(x_{1},x_{3},t)=[pA_{1}\cos(px_{3})-ikB_{2}\cos(qx_{3})]\times \exp[i(kx_{1}-\omega t)] \end{array}$$

Each group of solutions above consists of the traveling wave sections along the *x*_1_ direction and the standing wave sections along the *x*_3_ direction. The traveling wave sections show the relation between the displacements and the ULW propagation distance. The standing wave section of *u*_1_ in Equation (4) contains only cosine functions of *x*_3_, which means *u*_1_ is symmetric about *x*_3_ = 0. By convention, the solution of Equation (4) expresses the symmetric mode (S mode) ULW. Similarly, the solution of Equation (5) expresses the antisymmetric mode (A mode) ULW. Similarly, by solving Equations (1) and (2), the expressions for the stresses *σ*_31_ and *σ*_33_ induced by the ULW can be determined. By inserting the expressions for the stresses *σ*_31_ and *σ*_33_ into the boundary conditions: *σ*_31_ = *σ*_33_ ≡ 0 (*x*_3_ = ±*h*), the ULW frequency dispersion equations can be determined:

(6) $$\begin{array}{ll} \text{S mode}: & \frac{\tan(qh)}{\tan(ph)}=-\frac{4k^{2}pq}{{\left(q^{2}-k^{2}\right)}^{2}} \end{array}$$

(7) $$\begin{array}{ll} \text{A mode}: & \frac{\tan(qh)}{\tan(ph)}=-\frac{{\left(q^{2}-k^{2}\right)}^{2}}{4k^{2}pq} \end{array}$$

**Figure 2.**
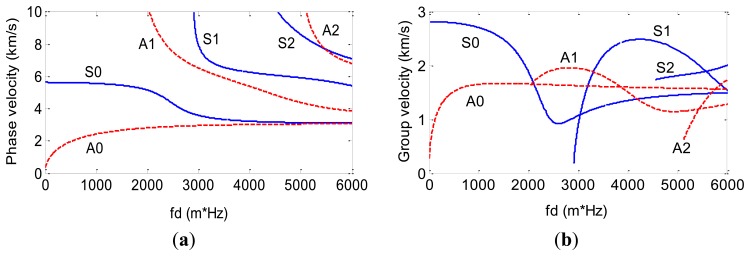
The ULW frequency dispersion curves of (**a**) *c_p_* and (**b**) *c_g_* for the 3 mm thick aluminum plate.

Based on Equations (6) and (7) and the definition of the ULW phase velocity (*c_p_* = *ω*/*k*) and group velocity (*c_g_* = d*ω*/d*k*), the frequency dispersion curves for the 3 mm thick aluminum plate used in the experiments of this paper are calculated as shown in Figure 2. The frequency dispersion curves show how *c_p_* and *c_g_* change with the product of the frequency and the thickness (*f*·*d*). Every curve in Figure 2 stands for a ULW mode.

Figure 2 shows that there are fewer ULW modes in the *f*·*d* range of 0∼1,650 Hz-m than in any other *f*·*d* range. In this range, only the A0 and S0 modes ULWs exist, and the changing rate of *c_g_* of the A0 mode ULW is larger than that of the S0 mode ULW. This means that A0 mode ULWs are more sensitive to the aluminum plate's changing thickness induced by the defects. Thus, the operation points of the new EMAT proposed in this paper are chosen from the *f*·*d* range of 0∼1,650 Hz-m, and the A0 mode ULW is used for the ULW TI.

### 2.2. The Contra-Flexure Structure and Transduction Principles of the New EMAT Coil

**Figure 3.**
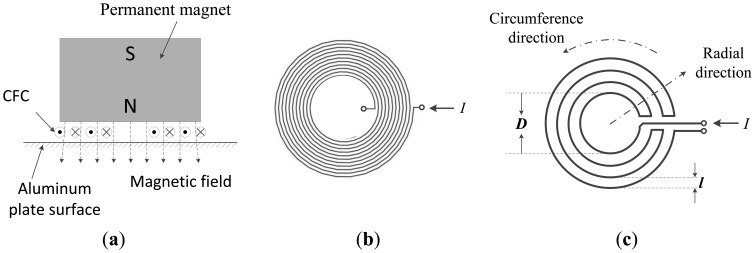
The structure and schematic diagrams of the omni-directional EMATs: (**a**) the structure of the new EMAT; (**b**) the traditional CWSC; and (**c**) the new CFC.

Both the traditional omni-directional EMAT with the CWSC and the new omni-directional EMAT proposed in this paper are composed of permanent magnets and coils as shown in Figure 3a. The permanent magnet is used to provide the bias magnetic field perpendicular to the surface of the aluminum plate. The structure of the traditional CWSC is shown in Figure 3b. The unique feature of the new EMAT is that the new CFC, shown in Figure 3c, is used as its transmitting and receiving coils. The CWSCs and the CFCs used in this paper are made using printed circuit board (PCB) technology. When a pulse current with a frequency *f* is switched on, every annular wire in the new CFC induces an eddy current field on the surface of the aluminum plate. In the bias magnetic field, under the effects of the Lorentz force, the eddy current field induces vibration in the aluminum plate along the radial direction of the annular wire. Next, the ULW pulses for different modes are transmitted and propagate along the radial direction. Because the vibration of the aluminum plate covers the entire circumferential direction, the ULW propagates omni-directionally.

Because the newly proposed coil has a contra-flexure structure, the eddy current fields induced by any two adjacent annular wires of the coil travel in the opposite directions so that the vibrations of the aluminum plate induced by the two eddy current fields have the opposite directions as well. Therefore, according to the relation between the displacement in aluminum plate and the ULW propagation distance shown in Equations (4) and (5), the ULW pulses of the mode of interest transmitted by any two adjacent annular wires could reinforce each other, while *l* (the distance between the two adjacent annular wires along the radial direction), *D* (the diameter of the innermost annular wire), the *f* (the frequency of the excitation pulse current), *λ*_int_ (the wave length of the ULW pulses of the mode of interest, excited by the two adjacent annular wires) and *c_p__*_int_ (the phase velocity of the ULW pulses of the mode of interest) satisfy the following matching relations:

(8) $$\{\begin{array}{l} f=\frac{c_{p\_\mathrm{int}}}{\lambda_{\mathrm{int}}}=\frac{c_{p\_\mathrm{int}}}{2l}\\ D=n\frac{\lambda_{\mathrm{int}}}{2}\;(n\;\text{isodd}\;\text{number},\;n>0) \end{array}$$

By contrast, the ULW pulses of every one of the remaining modes excited by the two adjacent annular wires weaken each other, because their wave-lengths do not satisfy the above matching relations. As a result, the ULW pulse of the mode of interest excited by the new EMAT with the CFC could be much purer. The *f* satisfying Equation (8) is defined to be the inherent operating frequency of the new EMAT. The principle behind receiving the ULW is the inverse process of transmitting the ULW. The traditional EMAT with the CWSC does not have any inherent operating frequency because the structure parameters of the traditional CWSC do not satisfy the above matching relation.

### 2.3. The ULW TI Method

Although various types of reconstruction algorithms can be used for ULW TI as mentioned above, the cross-hole tomography method is used in this paper to investigate the TI performances of the new EMAT, because this method is efficient and easy to use without requirement of rotating the transducer array [10]. Concretely, the rectangular area of the aluminum plate being imaged is subdivided into M × N small, equal meshes. The transmitting and receiving EMATs are arranged along the two sides of the area being imaged. The ULW rays pass through these meshes from different directions. When a ULW ray meets a mesh containing defects, such as corrosion thinning, the *c_g_* of the ULW changes with the thickness of the aluminum plate, which induces change in the TOF of the ULW ray. Thus, using the SIRT arithmetic, the distribution of the slowness (the reciprocal of the *c_g_*) and the defects of all the meshes can be determined by solving:

(9) $$\begin{array}{cc} T_{i}=\sum_{j=1}^{n}L_{ij}*S_{j} & \left(i=1,2,\ldots ,m\right) \end{array}$$

where *L_ij_* is the length of the *i-*th ULW ray in the *j-*th mesh; *T_i_* is the received real TOF of the *i-*th ULW ray, which is the projection data; and *S_j_* is the slowness of the *j-*th mesh.

## 3. Experiments and Results

### 3.1. The Experimental System

**Figure 4.**
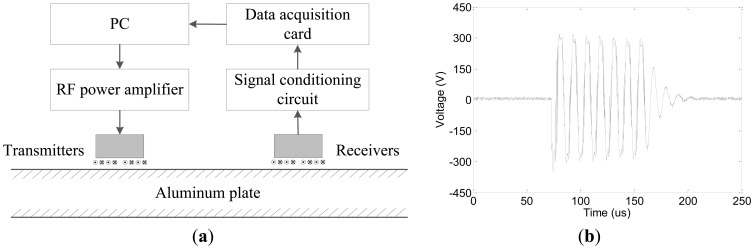
The schematic diagrams of experimental system. (**a**) The experimental system; and (**b**) the tone burst pulse excitation signal.

To verify the ability to transmit and receive a pure single A0 mode ULW, the accuracy of the TOF projection data extraction, the circumferential consistency of the projection data and the performances of the ULW TI for the new EMAT, several experiments were conducted using on a 3 mm thick aluminum plate. The schematic diagrams of the experimental system are shown in Figure 4a. There are two groups of EMATs: transmitters and receivers. The transmitters are excited by a RF power amplifier (AG1024) that is controlled by a personal computer (PC). The peak-to-peak amplitude of the tone-burst pulse excitation signal is approximately 300 V, and the periodicity is 7. The waveform of the tone-burst pulse excitation signal is shown in Figure 4b. The transmitted ULW passing pulse, the reflection pulses and the transmission defect pulses are detected by the receivers. The detected signals are filtered and amplified by a signal conditional circuit and subsequently collected by a high-speed DAQ card. Finally, the collected inspection data are sent to the PC to be calculated, analyzed, disposed of and used for the ULW TI.

The new CFC EMATs with an inherent operating frequency of 80 kHz and the traditional CWSC EMATs without any inherent operating frequency were used in these contrasting experiments to investigate the ability to transmit and receive a pure single A0 mode ULW, and a sampling frequency of 2.5 MHz was used. To investigate the circumferential consistency of the projection data and the performances of the new EMATs used for ULW TI, a higher operating frequency was needed to enhance the temporal resolution of the inspected waveforms and the accuracy of the projection data extraction. The higher frequency can increase the ULW attenuation, but in these experiments, the ULW propagation distance is short; therefore, the attenuation of the ULW can be ignored. Thus, the new CFC EMATs with a higher frequency of 290 kHz and a higher sampling frequency of 2.5 MHz were used for these experiments. Both 80 kHz and 290 kHz are in the ideal operating point range mentioned above where only A0 and S0 modes exist. Based on the frequency dispersion equations, the theoretical *c_g_* of the A0 mode ULWs with frequencies of 80 kHz and 290 kHz are 2,519 m/s and 3,265 m/s, respectively, in the 3 mm thick aluminum plate, while their theoretical *c_p_* are 1,446 m/s and 2,319 m/s with corresponding wavelengths of 1.8 cm and 0.8 cm, respectively.

### 3.2. A0 Mode ULW Transmitting and Receiving Experiment

**Figure 5.**
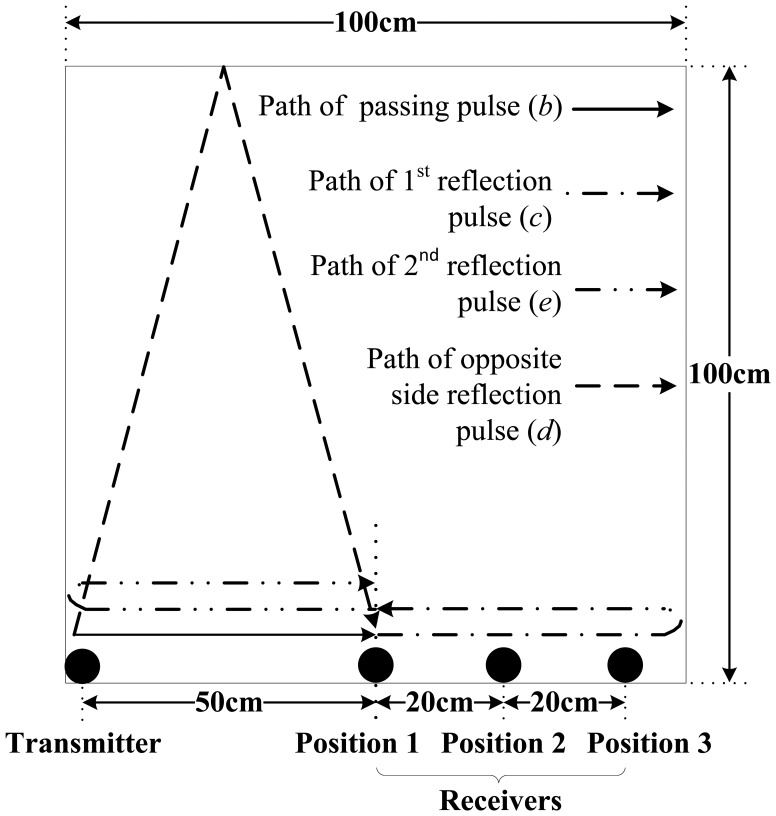
Schematic diagram of the EMAT positions and the propagation paths of the ULW pulses.

**Figure 6.**
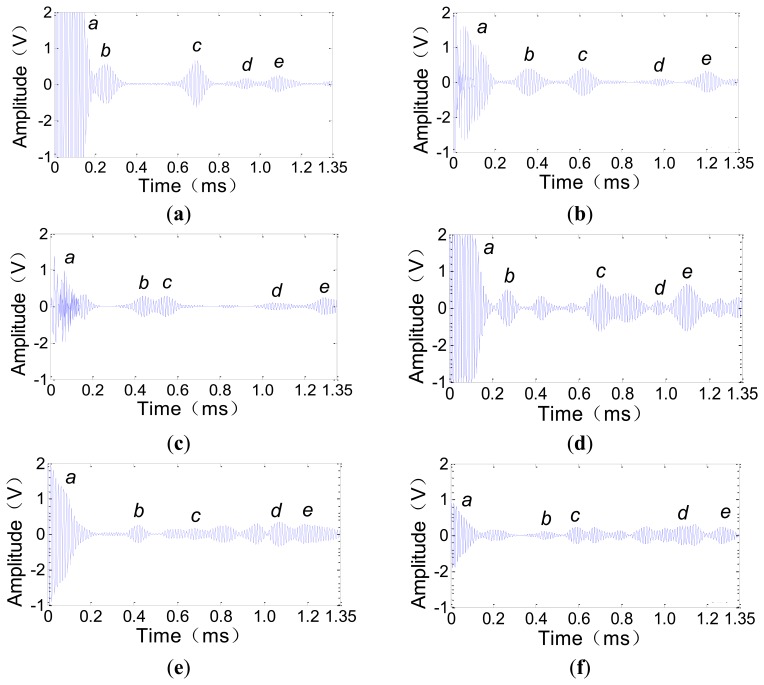
Detected waveforms of the A0 mode ULW transmitting and receiving experiments. (**a**) Using the new CFC EMATs, the receiver at position 1; (**b**) Using the new CFC EMATs, the receiver at position 2; (**c**) Using the new CFC EMATs, the receiver at position 3; (**d**) Using the traditional CWSC EMATs, the receiver at position 1; (**e**) Using the traditional CWSC EMATs, the receiver at position 2; (**f**) Using the traditional CWSC EMATs, the receiver at position 3.

The A0 mode ULW transmitting and receiving experiments were conducted using a 3 mm × 100 cm × 100 cm aluminum plate, as shown in Figure 5. The frequency of the tone-burst pulse excitation signal of the transmitter is 80 kHz. The experimental process and results are as follows. First, the new CFC EMATs are used as the transmitter and receivers. One transmitter is fixed at the corner, and three receivers are fixed at three different positions along one side of the aluminum plate as shown in Figure 5. After a single excitation of the transmitter, the three receivers are used to detect the passing ULW pulse and the reflection pulses. The waveforms detected by the receivers at positions 1, 2 and 3 are shown in Figure 6a–c, respectively. Next, four traditional CWSC EMATs are used in place of the four new CFC EMATs as the transmitter and receivers. After a single excitation of the transmitter, the waveforms detected by the receivers at the positions 1, 2 and 3 are shown in Figure 6d–f, respectively.

Figure 5 shows the propagation paths of the passing ULW pulse and the three side reflection pulses that are the first four pulses arriving at the receiver at position 1. These ULW pulses can be used to explain the corresponding signals in the detected waveforms in Figure 6a. In Figure 6a, signal *a* is the initial pulse, which is induced by the receiver coil in the space of the pulse excitation current; signal *b* is the first passing pulse; signal *c* and *e* are the 1st and 2nd side reflection pulses and *d* is the opposite side reflection pulse. The signals *a–e* in Figure 6b–f have the same meaning as the corresponding signals in Figure 6a.

First, the ULW transmitting and receiving results using the new CFC EMATs are qualitatively analyzed. By comparing Figure 6a with Figure 6b,c, it can be observed that in these detected waveforms, the position of the passing ULW pulse signal *b* moves backward when the distance between the receiver and the transmitter gradually increases. Additionally, the positions of the side reflection pulse signals *c*, *d* and *e* correspondingly move.

Second, to identify the generated ULW mode more accurately, a quantitative analysis is conducted. In Figure 6a, the *c_g_* of the generated ULW can be estimated as Δ*dis* (the propagation distance difference between the side reflection pulse, for example *c*, *d* or *e*, and the passing pulse *b*) divided by the corresponding Δ*t* (the time difference between the side reflection pulse, for example *c*, *d* or *e*, and the passing pulse *b*). By comparing the estimated *c_g_* and the theoretical *c_g_* of 2,519 m/s of the 80 kHz A0 mode ULW in the 3 mm thick aluminum plate, the mode of the generated ULW using the new CFC EMATs can be verified. Similarly, the detected waveforms in Figure 6b,c can be quantitatively analyzed. The results shown in Table 1 indicate that the estimated *c_g_* of the generated ULW is in good agreement with the theoretical *c_g_*. The maximum relative error *err =* |*c_g_* − *c_gth_*|/*c_gth_* = 5.5%. This means the new CFC EMATs could be used to transmit and receive A0 mode ULWs.

Finally, the performances of the ULW transmitting and receiving using the new CFC EMATs and the traditional CWSC EMATs can be compared using Figure 6a,d. These two figures are both waveforms detected by the receivers at position 1. In Figure 6a, the passing pulse *b* has a large amplitude and the waveform has a high signal-to-noise ratio (SNR). However, in Figure 6d, the passing pulse *b* has a smaller amplitude, and the waveform has a smaller SNR than Figure 6a. In addition, the ULW signals of other modes except for the A0 appear in Figure 6d, which do not appear in Figure 6a. By calculating *c_g_* as above, it can be verified that these new appearing signals are the S0 mode ULW signals. These new signals influence the identification of the A0 mode ULW signals and reduce the accuracy of the extraction of the projection data such as the amplitudes and TOFs of the A0 mode ULWs, especially when these S0 mode ULWs overlap with the A0 mode ULWs. For example, the amplitude of pulse *e* in Figure 6a should have been higher than that in Figure 6d just like what signal *b* does, but actually it is not, and the reason is as follows: according to the frequency dispersion equations, *c_g_* of the A0 and S0 mode ULWs in Figure 6d are, respectively, 2,519 m/s and 5,612 m/s. The propagation distances of the 2nd reflection pulse of the A0 mode ULW and the 5nd reflection pulse of the S0 mode ULW are 2.5 m and 5.5 m, respectively. Thus, the propagation time of these two ULW pulses are, respectively, 2.5 m/(2,519 m/s) = 992 μs and 5.5 m/(5,612 m/s) = 980 μs, and they are very approximate. This means the pulse *e* in Figure 6d is the superposition of the 2nd reflection pulse of the A0 mode ULW and the 5nd reflection pulse of the S0 mode ULW. The same results can be found by comparing Figure 6b,c with Figure 6e,f.

Based on the above analysis, the new CFC EMATs is seen to transmit and receive much purer A0 mode ULWs with a higher SNR than the traditional CWSC EMATs. These are helpful for the accurate extraction of the TOF projection data of A0 mode ULWs.

### 3.3. Circumferential Consistency Verification Experiment

**Figure 7.**
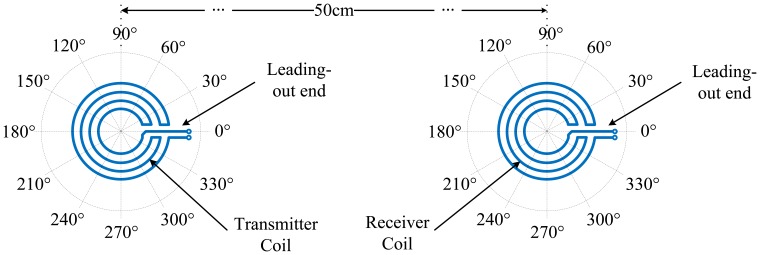
Schematic diagram of the EMATs positions and directions.

Two new EMATs of 290 kHz are used as the transmitter and receiver in the circumferential consistency verification experiments. The transmitter center is 50 cm away from the receiver center on the aluminum plate. For the convenience of the circumferential consistency investigation, the directions of the EMATs are defined as follows. Two polar coordinate systems are established on the aluminum plate, with the centers of the two EMAT coils as their respective origins, as shown in Figure 7. The polar coordinate systems are fixed relative to the aluminum plate. When the EMAT rotates about its center, its direction is defined as the angle at which the EMAT CFC leading-out end points towards.

**Figure 8.**
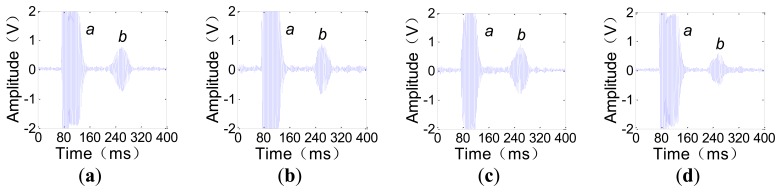
Detected waveforms when the receiver direction remains at 90° and the transmitter direction changes. (**a**) Transmitter direction is 30°; (**b**) Transmitter direction is 60°; (**c**) Transmitter direction is 90°; (**d**) Transmitter direction is 180°.

Because the TOF concerned by the TI is the time that the ULW takes to propagate from the transmitter to the receiver, only the initial pulse signal *a* and the passing pulse signal *b* are of interest in our experiments. For instance, when the receiver direction remains at 90° and the transmitter direction is 30°, 60°, 90° and 180°, the received waveforms are given as shown in Figure 8. For these waveforms, the initial pulse signal *a* and the passing pulse signal *b* are easily recognized.

**Figure 9.**
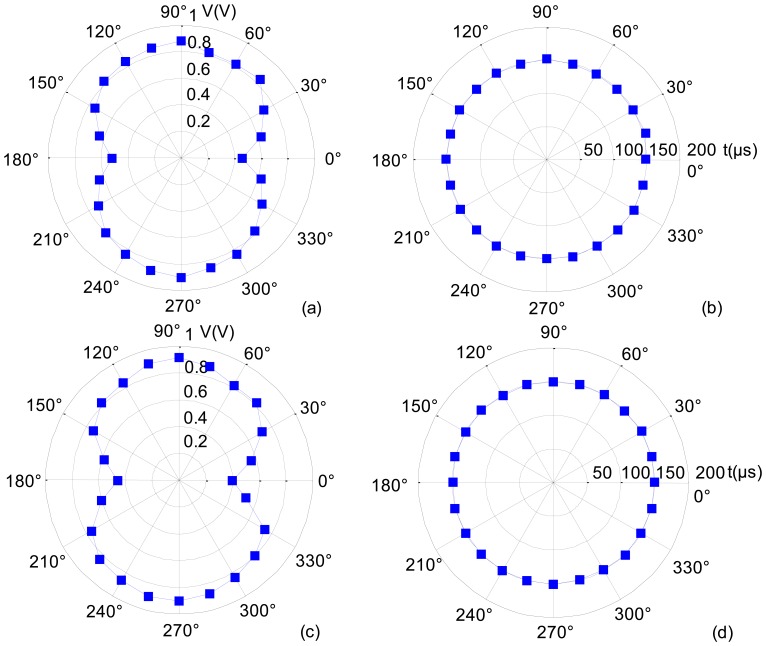
Results of the circumferential consistency verification experiments: (**a**) *V* of the different transmitter directions; (**b**) *t* of the different transmitter directions; (**c**) *V* of the different receiver directions; and (**d**) *t* of the different receiver directions.

To help facilitate the quantitative analysis, the time difference between signals *b* and *a* is denoted by *t*, which is the TOF of the A0 mode ULW, and the peak-to-peak amplitude of *b* is denoted by *V*. The experimental process and results are as follows. First, when the receiver direction remains at 90° and the transmitter direction varies by 360°, *V* and *t* are recorded. The results are shown in Figure 9a,b. In Figure 9a, *V* is smaller when the transmitter direction is approximately 0° or 180°. This is because the shape irregularity of the CFC leading-out end induces a distortion of the eddy current field in the aluminum plate, and the distortion of the eddy current field weakens the energy of the A0 mode ULWs that propagate along the CFC leading-out direction as well as in its opposite direction. Thus, when the CFC leading-out end is near the connecting line of the two EMATs, the energy of the received ULW pulse is weakened. The most direct evaluation of the circumferential inconsistency of *V* can be expressed as the relative difference between the maximum and minimum *V*:

(10) $$\text{d}V=\frac{V_{\max}-V_{\min}}{V_{\max}}\times 100\%$$

In Figure 9a, d*V* = 51.1%. In Figure 9b, *t* remains approximately constant when the transmitter direction varies by 360°. The evaluation index of the circumferential inconsistency of *t* is: d*t* = 4.8%. Next, when the transmitter direction remains at 90° and the receiver direction varies by 360°, *V* and *t* are recorded as shown in Figure 9c,d. The evaluation index of the circumferential inconsistency of *V* and *t* are found to be d*V′* = 57.0% and d*t′* = 3.0%.

The experimental results indicate that when the new CFC EMATs are used, because of the distortion of the eddy current field induced by the shape irregularity of the CFC leading-out end, the amplitude of the transmitted and received A0 mode ULW pulses distribute unevenly by 360°. However the circumferential consistency of the TOF projection data, which ULW TI is concerned with, is only slightly influenced by the distortion of the eddy current field.

### 3.4. ULW TI Experiment

**Figure 10.**
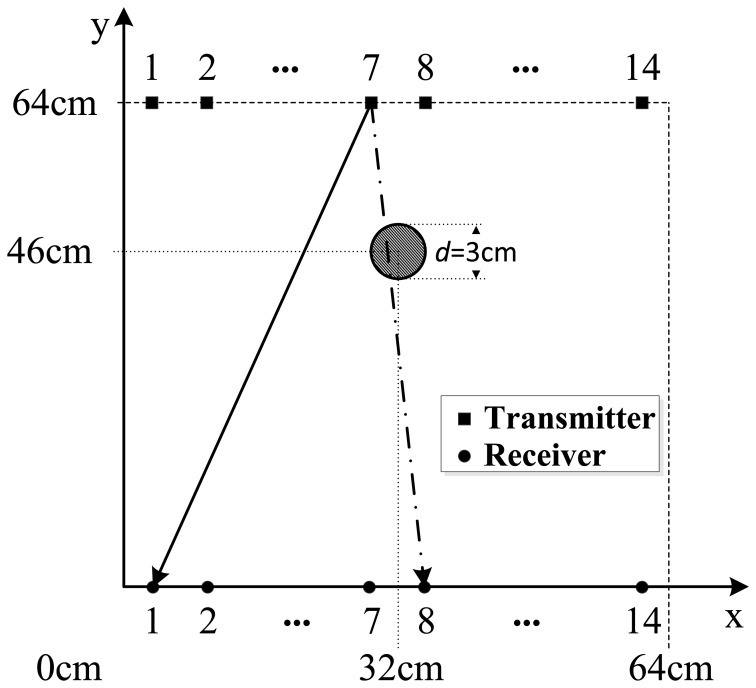
Schematic diagram of the ULW TI area.

To verify the ability of the ULW TI using the new CFC EMATs, the experiments are implemented on the 64 cm × 64 cm square area of the 3 mm thick aluminum plate, as shown in Figure 10. In this area and at the position (32 cm, 46 cm), there is a 2 mm deep artificial corrosion defect with a diameter of 30 mm. The square area being imaged is equably subdivided into 128 × 128 small meshes. Every small mesh is a 5 mm × 5 mm square. There are 14 transmitter positions on one side of this area, while there are 14 receiver positions on the opposite side. The diameters of these 290 kHz EMATs are 30 mm. The distance between every two adjacent EMATs is 45.7 mm. Additionally, the matrix *L_ij_* in Equation (9) is calculated.

**Figure 11.**
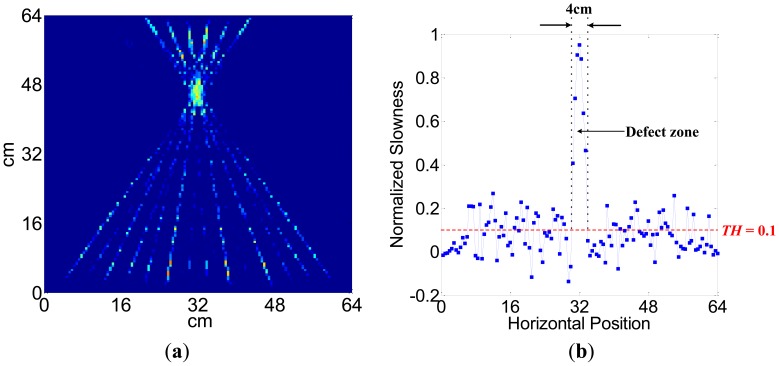
Results of the ULW TI experiment. (**a**) The TI result and (**b**) the slowness curve on the cross section at a position of y = 44 cm for the reconstructed image.

The process and results of the ULW TI experiment are as follows. In the first step, one transmitter is used to generate the A0 mode ULW and all the receivers are used to detect it. In the second step, the envelopes of the detected waveforms are taken through a Hilbert transformation [38]. In the third step, the TOF *t* that the passing pulse lags behind the initial pulse are extracted from the envelopes of the waveforms and recorded as the projection data. Next, the three steps are repeated until every transmitter has been used to generate the A0 mode ULW once. Next, the recorded TOFs of 196 ULW rays from the 14 pairs of transmitters and receivers are used as the input data *T_i_* of the SIRT arithmetic. The relaxation parameter is 0.1. After 100 iterations, the distribution of the ULW slowness of all the meshes is determined, and the image of the inspected area is reconstructed, as shown in Figure 11a. To analyze the reconstructed image, the normalized slowness curve on the cross section at the position y = 46 cm for the reconstructed image is extracted and shown as Figure 11b. Based on the slowness curve, it can be noted that the value of the slowness changes abruptly at the defect zone. The size of the defect can be estimated by choosing a threshold *TH* = 0.1. The estimated size of the defect is approximately 4 cm, which is approximately the actual size of the defect.

**Figure 12.**
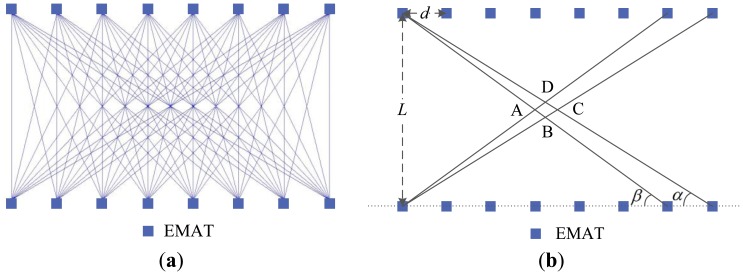
(**a**) The ULW ray trace pattern of the cross-hole method; (**b**) the schematic used for analyzing the relation between the resolution and the number of the EMATs for TI.

High construction resolution and fewer artifacts for ULW TI are always desired in practical applications [39]. In the following, the relation between the resolution and the number of the EMATs for ULW TI are analyzed. Figure 12 shows the ULW ray trace pattern of the cross-hole method and the schematic used for analyzing the relation mentioned above. Though, to ensure that the figures can be seen clearly, only eight pairs of EMATs are shown ether in Figure 12a or in Figure 12b, in practice there are 14 pairs. It can be noticed in Figure 12a that the density of the ULW rays in cross-hole method is uneven, especially on the side or around the corner of the reconstruction area. To get reconstruction fidelity, the region of interest should be set as close to the center of the reconstruction area as possible. In Figure 12b, the size of the central small quadrilateral zone ABCD can represent the average size of the meshes formed by the ULW ray paths, and can represent the minimum defect size that can be seen as well. Thus the reciprocal of the size of the zone ABCD can be defined as the reconstruction resolution. This size can be determined as follow:

(11) $$\{\begin{array}{l} S_{\text{ABCD}}=(\text{AC}\times \text{BD})/2=(\frac{d}{2}\times d\cdot \frac{\tan\alpha \tan\beta }{\tan\alpha +\tan\beta })/2\\ \tan\alpha =L/[(N-1)\times d]\\ \tan\beta =L/[(N-2)\times d] \end{array}$$

where *N* is the number of transducers along each line. Based on Equation (11), *S*_ABCD_ = *Ld*/[4(2*N* − 3)] can be determined. This result indicates that under the same distance *L*, a better reconstruction resolution can be obtained by increasing the number of the EMATs, according to the requirements of practical applications.

**Figure 13.**
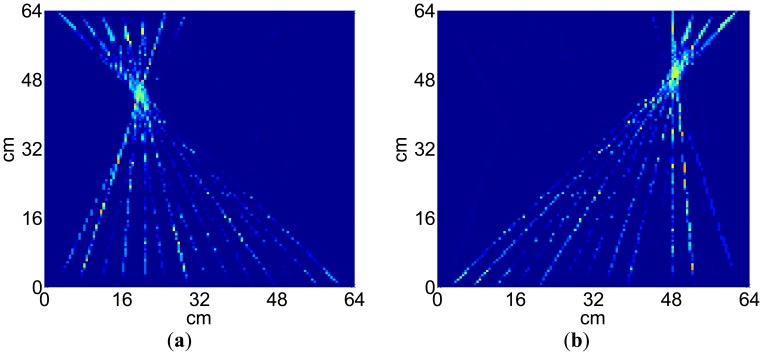
Experimental ULW TI results with the defect in locations (**a**) (20 cm, 44 cm) and (**b**) (49 cm, 49 cm).

Figure 13 shows the experimental ULW TI results with the defect in two different locations relative to the new EMAT array. The experimental conditions and the size of the defect are the same as above. The locations of the defect in Figure 12a and Figure 12b are (20 cm, 44 cm) and (49 cm, 49 cm), respectively. The results indicate that the new EMAT array can accurately locate and determine the size of the defect in different locations. The reconstruction shape of the defect is effected by the ULW rays passing by it. There is an elongated shape in ray direction for the defect around a corner of the reconstruction area, due to the so-called “shadow effect” caused by a low ray density there as shown in Figure 12b.

## 4. Conclusions

The non-contacting, omni-directional EMAT is an ideal choice for the ULW TI of the defects in metallic plates. In this paper, a new omni-directional ULW EMAT composed of a CFC and a permanent magnet was proposed. The theoretical analysis and experimental results indicate that by using the new CFC EMAT, a purer single A0 mode ULW with a higher SNR could be transmitted and received, and the extraction of the projection data for TI could be performed more accurately, compared to the traditional CWSC EMAT. Although the proposed CFC with the irregular shape induces a distortion of the eddy current field in aluminum plate, the circumferential consistency of the TOF projection data is only slightly influenced. The experimental results of imaging an artificial defect in 3 cm thick aluminum plate indicate that the new EMAT array has an acceptable ULW TI performances, and can accurately locate and determine the size of the defect when the defect is in different locations relative to the EMAT array. Moreover, according to the analyzed result, the reconstruction resolution can be improved by increasing the number of the new EMATs used, according to the requirements of practical applications.

## Figures and Tables

**Table 1.** Estimation of *c_g_* of the ULW transmitted and received by the new CFC EMATs.

**Signal**	**Figure 6a**			**Figure 6b**			**Figure 6c**		
		
	*c*	*d*	*e*	*c*	*d*	*e*	*c*	*d*	*e*
Δ*t* (μs)	420	645	810	247	595	833	84	541	825
Δ*dis* (m)	1.00	1.56	2.00	0.60	1.42	2.00	0.20	1.29	2.00
*c_g_* (m/s)	2381	2419	2469	2429	2387	2401	2381	2384	2424
*err*	5.5%	4.0%	2.0%	3.6%	5.2%	4.7%	5.5%	5.4%	3.8%
